# The spinal posture of computing adolescents in a real-life setting

**DOI:** 10.1186/1471-2474-15-212

**Published:** 2014-06-20

**Authors:** Yolandi Brink, Quinette Louw, Karen Grimmer, Esmè Jordaan

**Affiliations:** 1 Division of Physiotherapy, Department of Interdisciplinary Health Sciences, Faculty of Medicine and Health Sciences, Stellenbosch University, PO Box 19063, Tygerberg 7505, South Africa; 2 International Centre for Allied Health Evidence (iCAHE), University of South Australia, GPO Box 2471, Adelaide, SA 5000, Australia; 3 Department of Biostatistics, Medical Research Council of South Africa, PO Box 19070, Tygerberg 7505, South Africa; 4 Statistics and Population Studies Department, University of the Western Cape, Private Bag X17, Bellville 7535, South Africa

**Keywords:** Posture, Adolescent, Body weight, Computers

## Abstract

**Background:**

It is assumed that good postural alignment is associated with the less likelihood of musculoskeletal pain symptoms. Encouraging good sitting postures have not reported consequent musculoskeletal pain reduction in school-based populations, possibly due to a lack of clear understanding of good posture. Therefore this paper describes the variability of postural angles in a cohort of asymptomatic high-school students whilst working on desk-top computers in a school computer classroom and to report on the relationship between the postural angles and age, gender, height, weight and computer use.

**Methods:**

The baseline data from a 12 month longitudinal study is reported. The study was conducted in South African school computer classrooms. 194 Grade 10 high-school students, from randomly selected high-schools, aged 15–17 years, enrolled in Computer Application Technology for the first time, asymptomatic during the preceding month, and from whom written informed consent were obtained, participated in the study. The 3D Posture Analysis Tool captured five postural angles (head flexion, neck flexion, cranio-cervical angle, trunk flexion and head lateral bend) while the students were working on desk-top computers. Height, weight and computer use were also measured. Individual and combinations of postural angles were analysed.

**Results:**

944 Students were screened for eligibility of which the data of 194 students are reported. Trunk flexion was the most variable angle. Increased neck flexion and the combination of increased head flexion, neck flexion and trunk flexion were significantly associated with increased weight and BMI (p = 0.0001).

**Conclusions:**

High-school students sit with greater ranges of trunk flexion (leaning forward or reclining) when using the classroom computer. Increased weight is significantly associated with increased sagittal plane postural angles.

## Background

It is commonly accepted that good (or ‘neutral’) spinal postural alignment occurs when the centre of gravity of each spinal segment is vertically aligned with the segment below [1]. Postural control during sitting is the ability to generate muscular force in relation to body weight to control the internal relationship of body segments and to maintain equilibrium [2]. Normally adolescents display more anterior/posterior postural sway than medial/lateral sway during sitting [2]. The literature reports that neutral posture is associated with minimum strain on active and passive spinal structures (muscles and ligaments) [3-5]. It is therefore assumed that sitting with a neutral spinal posture reduces the likelihood of musculoskeletal pain symptoms [6,7]. One method of management of spinal pain is postural re-education, which is typically aimed at optimising a neutral alignment of spinal segments [8].

Adolescence is the time of critical skeletal growth in the vertebral column, making them particularly vulnerable to musculoskeletal pain if neutral sitting postures are not supported in schools [9-11]. Over the last five years, there has been a dramatic increase in the use of information computer technology by high-school students in South Africa [12]. Their vulnerability to musculoskeletal pain from spinal growth spurts may well be increased by exposure to poor sitting postures if they occur with computer use [13].

School-based programmes aimed at encouraging good sitting postures, with or without a computer, have not reported consequent reduction in musculoskeletal pain prevalence [14,15]. This may be due to a lack of clear understanding of how to describe ‘good’ adolescent posture, how best to measure it and whether there are thresholds or a range within which ‘good’ posture occurs, with cut-off values indicating abnormal (poor) posture. The aim of this paper is to describe the variability of five postural angles in a cohort of asymptomatic high-school students whilst working on desktop computers, in a typical South African school computer classroom and to report on the relationship between the postural angles and age, gender, height, weight and computer use.

## Methods

### Ethics

The Human Research Committee of Stellenbosch University approved the study (N08/08/209). Approval was obtained from the Western Cape Education Department (WCED), and written permission and informed consent was obtained from students, parents/legal guardians prior to data collection.

### Study design and population

A prospective 12 month longitudinal study was conducted. This paper reports on the baseline data from this study. The study population was Grade 10 high-school students in the Western Cape metropole of South Africa, aged 15–17 years, enrolled for the subject Computer Application Technology (CAT) at the beginning of the 2010 academic school year. Eligible schools participated in the Khanya project, a WCED initiative to increase computer literacy among educators and school students. The Khanya project was rolled-out in stages, thus eligible schools had to have fully functional computer rooms, with similar computer classroom furniture in terms of chairs, desks and computers.

### Eligibility

Students were excluded if: 1) they were not in the age range; 2) they had previously failed CAT and were repeating the subject; 3) they had been diagnosed with movement disorders or severe fixed skeletal abnormalities; and 4) they were symptomatic, complaining of upper quadrant musculoskeletal pain (UQMP) during the preceding month. UQMP was determined at pre-study eligibility screening, from questions in the Computer Usage Questionnaire (CUQ) [16,17]. UQMP refers to symptoms of soreness, tingling, burning and numbness pertaining to the occiput, cervical spine, upper extremities, the clavicles and the scapulae [18,19].

### Sample size

The data reported in this paper is a subset of the principle prospective study where UQMP was the outcome. The sample size was calculated (NCSS/PASS 11) [20] using the output of logistic regression models with pain as a binary response variable, on a continuous predictor (posture angles) and inflated to account for ineligibility (described above) and likely attrition over 12 months. Estimates concerning the likely prevalence of each ineligibility criteria had been identified in earlier work [16]. Sampling indicating that at least 821 students should be screened at baseline in order to attain 240 students at one-year follow-up, with 93% power at a 0.05 significance level [21,22].

### School sampling

Five schools in each of the four Education Management District Centres (EMDC)(total = 20 schools) were randomly selected, and were included in the study if they were co-educational, offered CAT and had more than 20 students likely to enroll in this subject in 2010. If a selected school did not comply with all these criteria, another school in the same EMDC was randomly selected, until the quota of eligible consenting schools had been filled.

### Screening eligible students

All Grade 10 high-school students in the selected schools who enrolled in CAT at the beginning 2010 were invited to join the study, and screened for eligibility. Potentially eligible students received informed consent letters (explaining study aims and procedures) to take home. Students were then excluded if they did not provide written informed consent, or were absent on day of testing.

### Measurement instruments

**Figure 1:**
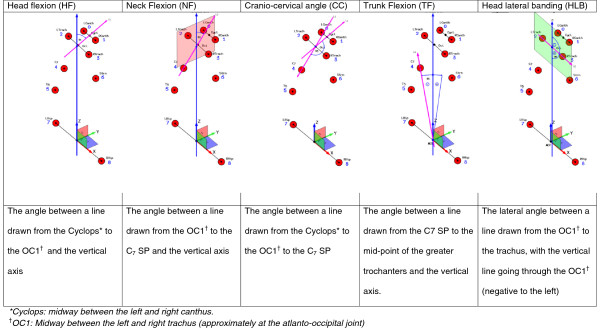
Schematic presentations and definitions of the five postural angles.

This project used a novel, portable, reliable and valid 3D Posture Analysis Tool (3D-PAT) which were described previously [23]. This tool is a basic implementation of stereovision, consisting of five cameras, a calibration object and designated software program. Its value was that it could be taken into school classrooms, and could capture accurate information on adolescents as they performed their computing tasks. The measurement instrument is configurable to allow for adaptation to various (spacious versus confined) classroom settings and dimensions [23]. This paper reports on the measurement of five postural angles as presented in Figure 1.

### Study procedure

#### *Preparation of classroom and students*

**Figure 2:**
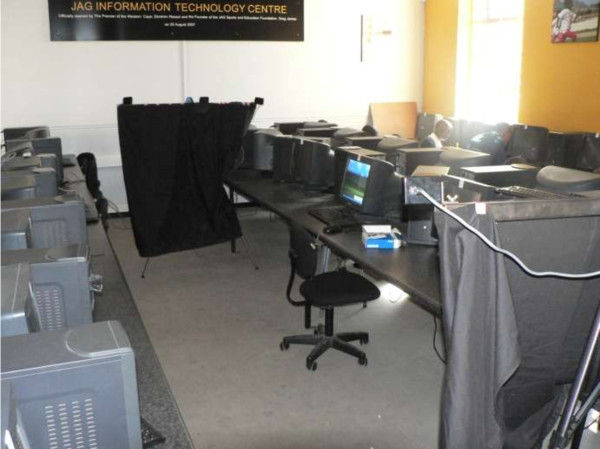
3D-PAT set-up in a school computer classroom.

Postural evaluation took place in the computer classroom. Two 3D-PAT camera units were positioned on each side of the student, facing the lateral aspect of the student as shown in Figure 2. The 3D-PAT was calibrated using a pyramid calibration object prior to each data capture per school. The measurement instrument’s set-up and calibration procedures are reported in detail elsewhere [23]. The 3D-PAT set-up was fixed at one computer workstation per school, with the computer monitor setting reflecting that provided in the school’s computer classroom during a normal class period.

**Figure 3:**
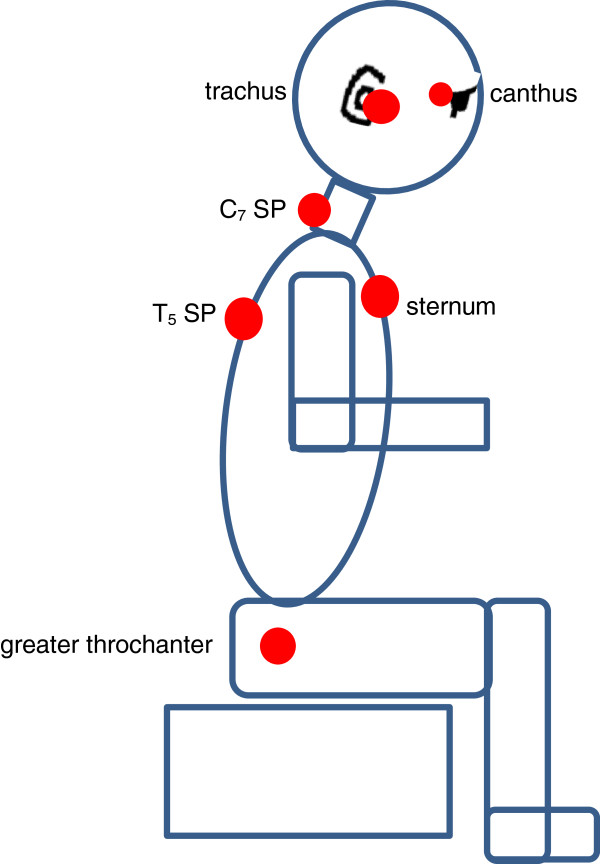
Schematic demonstration of the marker placement.

The students wore black t-shirts and grey school pants. Reflective markers were placed on nine anatomical landmarks i.e. the left and right canthus (the outer corner of the eye, where the upper and lower lids meet); the left and right trachus (the skin-covered cartilage in front of the meatus of the external ear); C_7_ spinous process (SP); T_5_ SP; both greater trochanters; and the superior border of the sternum, as illustrated in Figure 3. One researcher placed and removed all reflective markers, for consistency.

#### *Measurement of covariates*

Covariates included height, weight (also reported as BMI) and computer use. On the day of data capture, height was measured with a steel tape measure (Panamedic stature meter) mounted against the wall, and weight was measured with a calibrated digital scale (Terrailon Electronic Scale). As well as being used earlier for pre-study screening for UQMP, the CUQ was applied again on the day of data capture, to describe each student’s computer use at school and elsewhere, in terms of duration per session, frequency of usage, and total number of hours/week [17].

#### *Sitting postural evaluation*

Each student sat in front of the test computer, as they would usually do when performing a class activity. Students repeatedly typed a paragraph during testing. They typed for five minutes before the 3D-PAT captured postural alignment, and once data capture commenced, they were instructed to continue typing until the 3D-PAT had finished data capture [24,25]. Five minutes typing pre-capture was sufficiently long to encourage students to assume a relaxed posture, but short enough to minimise disruption of the academic programme of the school. Postural evaluation, from marker placement to removal, took approximately 7 minutes to complete.

### Data processing

One frame per camera was selected. To standardise the selection process, the frame closest to the 50th frame in which the student’s eyes were focused on the computer screen, were selected [26]. The marker selection procedure for the reflective markers on the students and on the calibration object were performed to reconstruct the 3D-coordinates of the reflective markers on the students, thus calculating the five postural angles [23]. Hours of weekly computer use at school, and elsewhere, were calculated separately, and then tallied as total computer use per week. BMI was calculated from height and weight [27].

### Statistical analysis

Summary statistics described the five postural angles as means, SD, medians, minimum and maximum values. Pearson correlation coefficients described the linear associations between pairs of postural angles. Computer use (defined above) was described as means, medians and SDs. Pearson correlation coefficients were calculated to describe the strength of linear associations between school, and elsewhere, computer use.

The associations between each postural angle, and each covariate, were first investigated with univariate linear regressions, and then with multivariate linear regressions adjusted for the significant covariates. Significance was p < 0.05. To investigate the impact of a combination of angles (describing a specific posture), factor analysis was performed to determine the latent constructs measured by the five postural angles (head flexion, neck flexion, cranio-cervical angle, trunk flexion and head lateral bend). A varimax rotation was also applied to identify orthogonal factors. The significant factor combination was then used in univariate and multivariate linear regression models, similarly to the individual angles.

## Results

### Sample composition

**Figure 4:**
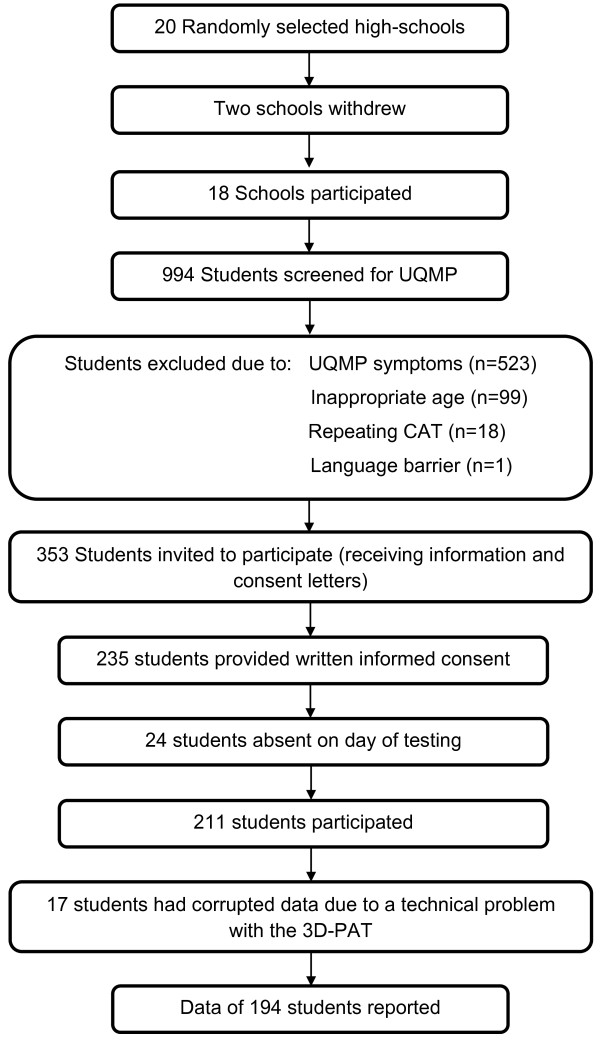
The sample composition at baseline.

Figure 4 summarises the sample composition.

### Covariates and sitting postural angles

Table 1 summarises the mean, SD, maximum, minimum and median values for age, height, weight, BMI and the five postural angles obtained from the 3D-PAT. The age and gender distribution of the participating students did not differ from those who were excluded from the study.

**Table 1:** The mean, median, SD, maximum and minimum values for height, weight, BMI and the five postural angles for the total group and by gender (n = 194)

	**Group (n = 194)**				**Males (n = 116)**	**Females (n = 78)**
	**Mean (SD)**	**Maximum**	**Minimum**	**Median**	**Mean (SD)**	**Mean (SD)**
Age	16.3 (0.5)	17.0	15.0	16.0	16.3 (0.5)	16.3 (0.5)
Height (m)	1.66 (0.1)	1.96	1.46	1.67	1.70 (0.1)	1.61 (0.1)
Weight (kg)	59.35 (13.1)	111.60	35.10	57.25	60.63 (13.0)	57.44 (13.1)
BMI	21.34 (3.9)	34.15	14.74	20.40	20.77 (3.5)	22.19 (4.3)
Head flexion (°)*	78.70 (8.4)	97.49	53.62	79,22	78.70 (8.5)	78.72 (8.2)
Neck flexion (°)^†^	61.55 (8.7)	92.64	31.87	61,14	61.71 (8.2)	62.26 (9.4)
Cranio-cervical angle (°)	161.62 (7.7)	178.81	141.67	161,27	161.72 (8.2)	161.44 (7.0)
Trunk flexion (°)^‡^	-9.53 (9.6)	18.84	-37.54	-9,06	-9.89 (9.7)	-9.02 (9.6)
Head lateral bend (°)^§^	-0.67 (5.1)	12.67	-15.26	-1,06	-0.61 (4.8)	-0.70 (5.4)

*Head flexion angles greater than 90° were obtained when the canthus was lower than the tragus.

^†^Neck flexion greater than 90° indicated that the tragus was lower than the level of the C_7_ SP.

^‡^If trunk flexion was positive, the C_7_ SP was positioned anterior to the greater trochanters, and the student had adopted a more flexed posture. If trunk flexion was negative, the C_7_ SP was positioned posterior to the greater trochanters, and the student had adopted a more extended posture.

^§^A negative value for head lateral bend indicated that the head was bent in the frontal plane to the left.

The correlations above 0.3 between paired individual angles were: head flexion with neck flexion (r = 0.504), trunk flexion (r = 0.326) and cranio-cervical angle (r = -0.480); neck flexion with trunk flexion (r = 0.593) and cranio-cervical angle (r = 0.399). Twenty-six of the 194 students sat leaning forward with a positive trunk flexion value (Lower quartile = 5.08, mean = 7.26, median = 6.54, Upper quartile = 7.87, minimum = 0.63, maximum = 18.84) and 168 in a reclined trunk position with negative trunk flexion value (Lower quartile = -6.76, mean = -12.13, median = -10.38, Upper quartile = -16.73, minimum = -1.25, maximum = -37.54). The inter-variability for head lateral bend was high compared to the mean (the SD was 7 times as large as the mean). Of the 194 students, 80 had a positive head lateral bend angle and 114 had a negative angle.

### Computer use

Descriptions of years of exposure to computer use, the duration of a computer session and the frequency of computer use per week at school and elsewhere, are reported in Additional file 1: Table S2. Computer exposure elsewhere, for example at home, indicated more years of computer experience and longer duration per session, but less frequency in computer use per week than was reported for at school computer use.

Additional file 1: Table S2 also presents the hours of computer use per week at school and elsewhere. The computer use at school was not highly correlated with the total weekly computer use (r = 0.21), whereas the computer use elsewhere was highly correlated with the total weekly computer use (r = 0.98). The latter high correlation implies that the majority of the total weekly computer use was used elsewhere. The school computer use was also not highly correlated with the computer use elsewhere (r = 0.03), implying that different children were spending computer time at school and elsewhere.

### Individual postural angles

Two response variables, trunk flexion-binary (indicating a positive or negative angle) and trunk flexion-numeric (measuring the size of the angle) were included in a multivariate regression analysis to be able to measure a possible association with either trunk flexion-binary and/or trunk flexion-numeric. The univariate linear regression analysis indicated that weight was significantly associated with head flexion, neck flexion and cranio-cervical angle; height with head flexion and neck flexion and BMI with neck flexion and cranio-cervical angle (Table 2). The multivariate linear regressions showed that height was marginally significantly associated with trunk flexion-binary (p = 0.055), thus the difference in height of the two groups were further investigated. The group sitting with a reclining trunk position (mean 1.67 cm ± 0.09) were 3 cm taller than the group sitting slightly forward (mean 1.64 cm ± 0.09). No significant associations were found between head lateral bend and any covariate.

**Table 2:** Univariate and Multivariate linear regression model demonstrating significant associations (p < 0.05) between individual angles, combined posture and covariates (weight, height and BMI)

	**Covariates**	**Estimate**	**SE**	**t - value**	**p value**
Head flexion	Weight	0.09	0.04	2.07	0.040*
	Height	14.62	6.52	2.24	0.026*
	BMI	0.15	0.15	1.02	0.311
Neck flexion	Weight	0.20	0.04	5.50	0.0001*
	Height	20.17	5.68	3.55	0.001*
	BMI	0.57	0.13	4.55	0.0001*
Cranio-cervical angle	Weight	0.10	0.04	2.54	0.012*
	Height	4.27	6.15	0.69	0.488
	BMI	0.41	0.14	3.03	0.003*
Trunk flexion-binary	Weight	-0.01	0.01	-0.09	0.930
	Height	-4.31	2.24	-1.92	0.055
	BMI	0.05	0.05	1.01	0.313
Trunk flexion-numeric	Weight	0.03	0.04	0.79	0.430
	Height	-1.30	5.54	-0.23	0.815
	BMI	0.13	0.127	1.09	0.277
Factor 1	Weight	0.023	0.004	5.04	0.0001*
	Height	2.505	0.711	3.52	0.001*
	BMI	0.064	0.016	4.00	0.0001*
Factor 2	Weight	0.005	0.005	1.00	0.319
	Height	-0.339	0.8	-0.42	0.672
	BMI	0.032	0.018	1.77	0.079

*Significant associations ρ < 0.05.

Gender, age and computer use were not significantly associated with the individual angles and the statistics are therefore not reported in the table, however they are presented in Additional file 2.

The associations between each postural angle, and each covariate, adjusted for age, gender and computer use, indicated that weight was significantly associated with neck flexion, head flexion and cranio-cervical angle; height with neck flexion and head flexion and BMI with neck flexion and cranio-cervical angle (Table 3). However, the marginally significant association between BMI and trunk flexion-binary was not retained after controlling for possible confounders.

**Table 3:** Multiple linear regression model estimates demonstrating significant associations (p < 0.05) between individual angles, combined posture and covariates, adjusted for age, gender and computer hours

	**Covariates**	**Estimate**	**SE**	**t - value**	**p value**
Head flexion	Height	18.1	7.88	2.29	0.023
	Weight	0.1	0.04	2.05	0.041
Cranio-cervical angle	Weight	0.1	0.04	2.45	0.016
	BMI	0.4	0.12	3.09	0.002
Neck flexion	Height	24.7	6.84	3.61	0.0004
	Weight	0.2	0.04	5.39	0.0001
	BMI	0.6	0.13	4.72	0.0001
Factor 1	Height	3.1	0.86	3.57	0.0005
	Weight	0.01	0.01	4.94	0.0001
	BMI	0.1	0.02	4.19	0.0001

### Combinations of postural angles

Two important factors were identified from the combination of the five angles. Factor one was a linear combination with high loadings (>40) for head flexion (56), neck flexion (97) and trunk flexion (60), which in combination explained 55% of the variability. Factor two was a linear combination with high loadings (>40) for head flexion (-80) and cranio-cervical angle (91), explaining 46% of the variability in the five angles.

When these two factors were considered as outcomes for regression modelling, weight, height and BMI were significantly associated with factor one, as shown in Table 2. These variables remained significant associates of factor one after adjusting for age, gender and computer use (Table 3). Factor one is interpreted as the weighted increase of head flexion, neck flexion and trunk flexion values/angles. For trunk flexion this means an increase from greater negative values/angles (leaning backwards) towards more positive trunk flexion values/angles (leaning forwards). There were no significant predictors when modelling Factor 2, which was interpreted as the weighted increase in head flexion and decrease in the cranio-cervical angle values.

## Discussion

The study reports new information that angles producing movement in the sagittal plane were either individually or in combination associated with height, weight and BMI. Firstly the individual angle, such as cranio-cervical angle, is an inter-segmental angle and closely related to head flexion and neck flexion. If cranio-cervical angle increases, this means that either head flexion decreased, or neck flexion increased. Keeping that in mind, and also considering the magnitude of significance for the associations between head flexion and weight (p = 0.041) and height (p = 0.023) compared to that for associations between neck flexion and height (p = 0.0004), weight (p = 0.0001) and BMI (p = 0.0001), it appears that the significant associations for neck flexion with weight and BMI are the most important and consistent findings. Thus heavier students have more neck flexion when working on desktop computers in their school computer classroom, than lighter students.

Secondly, weight might also be the more prominent contributor to the associations between the combination posture (head flexion, neck flexion and trunk flexion) and weight, height and BMI for the same reasons. When considering the change in trunk flexion, from an maximum negative angle (-37.54°) to a maximum positive angle (18.84°) as head flexion and neck flexion increases, it appears that these students sat with a more neutral trunk alignment as weight increased. This is reinforced by the upper quartile for trunk flexion being -4.65°. In contrast, Burgess-Limerick et al., (2000) found a reclined trunk position to be correlated with increased neck flexion and head flexion in order to accommodate for the height of the computer monitor [28]. Since the computer height and chair placement were according to student preference, and represented the student’s habitual classroom posture, it seems that students assumed postures due to intrinsic mechanisms and not to account for the height of the computer monitor [24]. Our study did not report on the height of the computer screen in relation to the anthropometrics of the students, however, Van Niekerk et al., (2013) reported that 89% of computer classroom chairs do not match the anthropometrics of adolescents from the same study population [29]. Considering computer screen height in further research seems sensible to better understand individual posture related to computer use.

The findings of our study concur in part with King et al., (2012) who report that increased BMI negatively impacts on postural control of adolescents in unstable positions i.e. one leg standing and moving from sit to stand [30]. These researchers did not examine postural control during sitting. Increased postural sway in standing and decreased postural stability during the initial phase of gait have also been noted for obese adolescents [31,32]. However an increased BMI in this study does not mean that students necessarily fall within the overweight or obese categories, as 75% of female and male students had BMI scores less than 24.04 and 22.20 respectively. Therefore the increase in sagittal plane postural angles, leading to a slightly forward-leaning and flexed head-on-neck posture might be an intrinsic mechanism due to decreased postural control and increased postural sway in the sagittal plane related solely to weight.

Hansen et al., (2008) found that increased sedentary behaviour rather than decreased physical activity was associated with increased BMI in adolescents [33]. In our study sedentary behaviour is reflected by the amount of weekly computer use but we found no significant correlation between computer use and BMI [r = 0.01 (0.887)] or weight [r = 0.081 (0.245)].

Four of the five postural angles (head flexion, neck flexion, cranio-cervical angle and trunk flexion) compare well with previous studies using the same angle definitions [25,34-36]. The only previous research to measure head lateral bend, as defined in this study, was our earlier study assessing the psychometric ability of the 3D-PAT to measure head lateral bend, where we reported a mean angle of 4.3° (±4.2) in similarly-selected adolescents [23]. The 4.97° difference in head lateral bend between the two studies might be due to the previous study having a small sample size (n = 24) and being laboratory based, whereas this study included 194 participants and reflected real-life sitting posture.

No significant relationship between gender and posture was reported in this study. This could be attributed to the fact that gender differences in adolescents’ sitting posture have been noted for lumbar and pelvic tilt angles which have not been measured in this study [37-39]. Our study also found no significant difference in weight between male and female adolescents (Table 1).

No association between computer use and posture was reported in this study. Straker et al., (2011) reported on postural differences between adolescent computer and non-computer users and found computer users to have increased neck flexion and increased pelvic tilt (not measured in this study) significantly associated with increased computer use [40]. Straker et al., (2007) reported increased computer use to be significantly associated with head flexion and neck flexion especially for boys and increased lumbar lordosis for girls [34]. The computer use reported in both studies reflected at school and elsewhere use and the posture was assessed in a laboratory setting without the subject facing a computer display, as done in this study.

### Study limitations

The loss of postural data (n = 17) could have influenced the magnitude and the number of observed associations between sitting posture and its covariates.

## Conclusions

This paper found trunk flexion to be the most variable postural angle measured and increased neck flexion was significantly associated with increased weight.

## Abbreviations

WCED: Western Cape education department; CAT: Computer application technology; UQMP: Upper quadrant musculoskeletal pain; CUQ: Computer usage questionnaire; 3D-PAT: 3D posture analysis tool; SP: Spinous process.

## Competing interests

The authors declare that they have no competing interests.

## Authors’ contributions

All the authors contributed to the conception and design of the study, one author (YB) acquired the data, two authors analysed the data (EJ and YB), all the authors contributed to the interpretation of the data, three authors (YB, QL and KG) drafted the manuscript and all the authors critically appraised the content of the manuscript. All authors read and approved the final manuscript.

## Pre-publication history

The pre-publication history for this paper can be accessed here:

http://www.biomedcentral.com/1471-2474/15/212/prepub

## Supplementary Material

Additional file 1: Table S2 Years of exposure, duration per session and frequency of weekly computer use at school and elsewhere and the mean, SD, maximum, minimum and median values for weekly computer use at school and elsewhere (n = 194). Click here for file

Additional file 2 Univariate and Multivariate linear regression model demonstrating associations between individual angles, combined posture and covariates (age, gender and computer use). Click here for file
