# Supplementary material for: The spinal posture of computing adolescents in a real-life setting
Source: BMC Musculoskelet Disord. 2014 Jun 20;15:212. doi: 10.1186/1471-2474-15-212 (PMC4094537; doi:10.1186/1471-2474-15-212)
Supplement: Additional file 1: Table S2 — Years of exposure, duration per session and frequency of weekly computer use at school and elsewhere and the mean, SD, maximum, minimum and median values for weekly computer use at school and elsewhere (n = 194). [file 1471-2474-15-212-S1.docx]

Table S2: Years of exposure, duration per session and frequency of weekly computer use at school and elsewhere and the mean, SD, maximum, minimum and median values for weekly computer use at school and elsewhere (n = 194)

| **At school** | | | | | | **Elsewhere** | | | |
| --- | --- | --- | --- | --- | --- | --- | --- | --- | --- |
| **Years of exposure** | | | | | | | | | |
|  | | **Frequency (n)** | **Percentage (%)** | |  | | **Frequency (n)** | | **Percentage (%)** |
| **< 1 yr.** | | 109 | 56.2 | | **< 1 yr.** | | 53 | | 27.5 |
| **2 yrs.** | | 30 | 15.5 | | **2-3 yrs.** | | 50 | | 25.9 |
| **3 yrs.** | | 25 | 12.9 | | **4 yrs.** | | 25 | | 13.0 |
| **> = 4 yrs.** | | 30 | 15.5 | | **> = 5 yrs.** | | 65 | | 33.7 |
| **Duration per session** | | | | | | | | | |
|  | | **Frequency**  **(n)** | **Percentage (%)** | |  | | **Frequency (n)** | | **Percentage (%)** |
| **<30 min** | | 7 | 3.6 | | **<30 min** | | 51 | | 26.4 |
| **45 min** | | 157 | 80.9 | | **1 h** | | 80 | | 41.5 |
| **1 h** | | 25 | 12.9 | | **2-3 h** | | 51 | | 26.4 |
| **1 ½ h** | | 4 | 2.1 | | **> = 4 h** | | 11 | | 5.7 |
| **2/+ h** | | 1 | 0.5 | |  | |  | |  |
| **Frequency per week** | | | | | | | | | |
|  | | **Frequency**  **(n)** | **Percentage (%)** | |  | | **Frequency (n)** | | **Percentage (%)** |
| **Once or less** | | 3 | 1.6 | | **Once or less** | | 21 | | 10.9 |
| **Twice** | | 2 | 1.0 | | **Twice** | | 42 | | 21.9 |
| **Three times** | | 19 | 9.8 | | **Three times** | | 34 | | 17.7 |
| **Four times** | | 43 | 22.2 | | **Four times** | | 16 | | 8.3 |
| **Five times** | | 127 | 65.5 | | **Five times** | | 79 | | 41.2 |
|  | **Computer usage at school per week (h)** | | | **Computer usage elsewhere per week (h)** | | | | **Total computer usage per week (h)** | |
| **Mean** | 3.55 | | | 5.36 | | | | 8.91 | |
| **SD** | 0.9 | | | 4.9 | | | | 5.1 | |
| **Minimum** | 0.75 | | | 0 | | | | 2.0 | |
| **Maximum** | 10.0 | | | 20.0 | | | | 23.75 | |
| **Median** | 3.75 | | | 3.00 | | | | 7.75 | |
