# Supplementary material for: The spinal posture of computing adolescents in a real-life setting
Source: BMC Musculoskelet Disord. 2014 Jun 20;15:212. doi: 10.1186/1471-2474-15-212 (PMC4094537; doi:10.1186/1471-2474-15-212)
Supplement: Additional file 2 — Univariate and Multivariate linear regression model demonstrating associations between individual angles, combined posture and covariates (age, gender and computer use). [file 1471-2474-15-212-S2.docx]

Appendix 1: Univariate and Multivariate linear regression model demonstrating associations between individual angles, combined posture and covariates (age, gender and computer use)

|  | **Covariates** | **estimate** | **SE** | **t - value** | **p value** |
| --- | --- | --- | --- | --- | --- |
| **HF** | age | -0.24 | 1.17 | -0.21 | 0.834 |
|  | gender | -0.76 | 1.15 | -0.66 | 0.512 |
|  | hrs. school* | 0.11 | 0.60 | 0.18 | 0.854 |
|  | hrs. else^†^ | -0.13 | 0.12 | -1.14 | 0.254 |
|  | total hrs.^‡^ | -0.12 | 0.11 | -1.08 | 0.280 |
| **NF** | age | 0.68 | 1.03 | 0.66 | 0.51 |
|  | gender | -0.78 | 1.02 | -0.76 | 0.447 |
|  | hrs. school^*^ | 0.02 | 0.64 | 0.03 | 0.976 |
|  | hrs. else^†^ | -0.05 | 0.1 | -0.50 | 0.616 |
|  | total hrs.^‡^ | -0.05 | 0.1 | -0.49 | 0.626 |
| **CC** | age | 0.54 | 1.09 | 0.49 | 0.623 |
|  | gender | -0.49 | 1.08 | -0.45 | 0.65 |
|  | hrs. school^*^ | 0.96 | 0.56 | 1.73 | 0.086 |
|  | hrs. else^†^ | 0.06 | 0.11 | 0.51 | 0.608 |
|  | total hrs.^‡^ | 0.09 | 0.11 | 0.83 | 0.406 |
| **TF binary** | age | -0.16 | 0.311 | -0.52 | 0.602 |
|  | gender | 0.54 | 0.383 | 1.40 | 0.163 |
|  | hrs. school^*^ | 0.04 | 0.042 | 0.88 | 0.379 |
|  | hrs. else^†^ | 0.11 | 0.129 | 0.83 | 0.408 |
|  | total hrs.^‡^ | 0.03 | 0.04 | 0.65 | 0.514 |
| **TF numeric** | age | -1.46 | 1.02 | -1.44 | 0.152 |
|  | gender | -0.27 | 0.992 | -0.27 | 0.789 |
|  | hrs. school^*^ | 0.17 | 0.096 | 1.81 | 0.071 |
|  | hrs. else^†^ | 0.01 | 0.52 | 0.02 | 0.987 |
|  | total hrs.^‡^ | 0.18 | 0.099 | 1.84 | 0.066 |
| **Factor 1** | age | 0.075 | 0.129 | 0.59 | 0.559 |
|  | gender | -0.101 | 0.127 | -0.80 | 0.427 |
|  | hrs. school^*^ | -0.01 | 0.08 | -0.07 | 0.942 |
|  | hrs. else^†^ | -0.007 | 0.013 | -0.57 | 0.569 |
|  | total hrs.^‡^ | -0.007 | 0.013 | -0.57 | 0.570 |
| **Factor 2** | age | 0.066 | 0.141 | 0.47 | 0.641 |
|  | gender | 0.005 | 0.139 | 0.04 | 0.971 |
|  | hrs. school^*^ | 0.08 | 0.09 | 0.87 | 0.383 |
|  | hrs. else^†^ | 0.012 | 0.014 | 0.87 | 0.384 |
|  | total hrs.^‡^ | 0.014 | 0.014 | 0.99 | 0.321 |

*Hours of weekly school computer use

^†^Hours of weekly computer use elsewhere

^‡^Total hours of weekly computer use
